# A GPT-reinforced social robot for patient communication: a pilot study

**DOI:** 10.3389/fdgth.2025.1653168

**Published:** 2026-01-27

**Authors:** Jan-Willem J. R. van 't Klooster, Michela Capasso, Daan van Gorssel, Elvis Vrolijk, Giorgio Rettagliata, Demy Gerritsen, Mirjam Hegeman, Emanuele Tauro, Enrico Gianluca Caiani, Harald E. Vonkeman

**Affiliations:** 1 Behavioural Management and Social Sciences, University of Twente, Enschede, Netherlands; 2 Dipartimento di Elettronica, Informazione e Bioingegneria, Politecnico di Milano, Milano, Italy; 3 Department of Rheumatology and Clinical Immunology, Medisch Spectrum Twente, Enschede, Netherlands; 4 IRCCS Istituto Auxologico Italiano, San Luca Hospital, Milan, Italy

**Keywords:** GPT, osteoarthritis, patient communication, social robot, UEQ

## Abstract

**Problem:**

Quality healthcare requires effective patient communication. However, lack of personnel and increasing demands on healthcare professionals (HCPs) create a need for innovative solutions that enhance accessibility and delivery of information to patients.

**Goal:**

We propose an innovative method to convey treatment and disease information using an Artificial Intelligence (AI)-driven social robotic physical interface. The aim of this study is to develop and test the feasibility of using a social robot that can convincingly provide health information in patient dialogues within clinical practice, to support patient communication and information exchange.

**Methods:**

This paper sets out the architectural approach of an AI-reinforced social robot connected to whitelisted validated clinical sources using a Generative Pre-training Transformer (GPT)-based Large Language Model (LLM). We describe experimental results in a lab-based pilot feasibility study, and then highlight related results for user experience in clinical practice implementation for an osteoarthritis (OA) use case, in which the robot answers osteoarthritis-related questions. Results were obtained after end-user engagement using the User Experience Questionnaire (UEQ) and semi-structured interviews.

**Results:**

UEQ results were obtained in a lab-based pilot test (*n* = 20) and with OA patients (*n* = 21) and healthcare professionals (*n* = 7). Above average/good attractiveness, perspicuity and stimulation were reported in the pilot test; novelty was excellent, yet dependability and efficiency were reported below average. In the clinical setting, Patient UEQ score resulted in mean 2.13 with values ranging from 1.7 to 2.5, indicating a positive trend in efficiency, inventiveness and acceptability. HCPs UEQ scores reached mean 1.89, with all values above 1 except for excitement of usage, which scored 0.8 (SD 1.3). Semi-structured interviews added in-depth enrichment of the data.

**Conclusion:**

In summary, this paper demonstrates the feasibility of implementing a GPT-reinforced social robot for patient communication in clinical practice.

## Introduction

Quality healthcare requires effective patient communication (1). However, lack of personnel and increasing demands on healthcare professionals (HCPs) create a need for innovative solutions that enhance accessibility and delivery of information to patients.

Therefore, we propose an innovative method to convey disease and treatment information. By using an Artificial Intelligence (AI)-driven approach and a social robotic physical interface, we hypothesized that communication could be adapted beyond pre-programmed messages and strategies, personalized and empathized to individual needs beyond screen-based applications, thus providing a multimodal communication experience. The research question addressed is whether it is possible to develop an AI reinforced social robot that answers patients' questions in such a way that it could have added value in clinical practice. Therefore, the aim of this study is to develop and evaluate a social robot that can convincingly provide patient information in patient dialogues in clinical practice, to support patient communication and information exchange. To this end, this paper sets out the architectural approach of the developed AI-reinforced social robot, it describes the obtained experimental results in a pilot feasibility study, and it highlights related results of user experience in a clinical practice implementation at a collaborating hospital institution. As a relevant use case, osteoarthritis (OA) was selected: OA represents a patient group with a complex chronic disease, where information and regular guidance are needed.

### Osteoarthritis (OA)

OA is a common chronic, progressive and disabling joint disease that results from degeneration of joint cartilage and underlying bone, causing progressive joint pain, stiffness and loss of motion. OA is one of the leading causes of disability in the world, affecting 1 in 7 adults in the Western world. Guidelines indicate that treatment should encompass both pharmacological and non-pharmacological management strategies, such as use of painkillers and lifestyle modification (2). However, many patients continue to suffer from daily complaints and limitations and therefore have high need for frequent guidance. It is beyond the scope of this article to provide a detailed overview of OA. For more information, the reader is referred to (2).

In the present study, this disease was chosen because of its high prevalence, chronicity, complexity and high patient guidance needs, leading to frequent hospital visits. This implies that there is a potential and significant gain in disease management, both at the societal and at the patient level, if novel technology could be utilized for increasing patient empowerment.

### Communication

Appropriate communication on health literacy and therapy adherence is essential for the success of treatment, both from a personal and societal perspective. This is particularly true for OA (3) but also applies to other medical conditions. In patient communication, it is important to check, maintain and increase health literacy, and promote therapy adherence. These factors play a crucial role in treatment outcome and overall success (4, 5).

When healthcare professionals do not have sufficient time to provide explanations (repeatedly) at an appropriate language level, social robotics could offer a scalable solution by engaging patients in accessible conversation and delivering understandable information (6, 7).

### Social robots

Social robots are physically embodied artificial agents designed to interact with users through verbal and nonverbal cues via a social interface. Robots' social features, which mimic the behaviour and appearance of a living being, lead users to perceive them as social entities (8).

Social robots are emerging as promising tools in the ICT landscape, offering the ability to interact naturally with humans, convey basic emotions and assist with communication tasks. By integrating Artificial Intelligence (AI), these robots can further adapt to individual and organisational needs, thus providing personalized support and relevant information.

Practitioners and researchers are increasingly paying attention to the use of social robots in healthcare (9). This growing interest is primarily due to the potential of social robots to address challenges posed by an ageing population and the rising labour shortages in the healthcare sector (10, 31). It is largely attributed to their potential to aid in the social management of health across various dimensions, ranging from assisting with medication schedules (11) to providing companionship to patients (12).

To fully realise these benefits, users must be willing to accept both the use of the robot for those tasks and the resulting outcomes. Establishing trust in the robot is essential for achieving this goal (13). Trust related to technological tools/agents can be defined as “*the attitude that an agent will help achieve an individual's goals in a situation characterized by uncertainty and vulnerability*” (14, p. 54). Indeed, trust in the robot is important because it also affects the willingness to accept the robot-provided outcome (42).

The concept of trust is multifaceted; initial perceptions influence the trustworthiness of the counterpart (43), but these impressions of the robot may change over time with subsequent interactions (15). These continuous interactions, in fact, lead to a continuous calibration of the trust placed in the robot itself so to arrive at a balance between expectations about the robot's performance and the robot's actual capabilities (16).

The specific characteristics of social robots are a novelty within the healthcare field and may influence this trust process differently to those of similar technologies, such as chatbots or virtual agents. For simple tasks, humans tend to trust physically present robots more than virtual agents, due to their enhanced social features, such as the ability to make gestures (17). Indeed, the physical presence and embodied interaction of robots (such as nodding while listening, eye gaze and other verbal behaviours) could provide more empathetic interactions and consequently elicit higher affective trust (18). However, when making moral decisions regarding healthcare, people tend to trust human nurses more than robots, even when social robots are perceived as competent. This preference reflects a more positive judgment of human nurses compared to robots (19). In such cases, it is evident that the social presence of the robot and the context of use could influence the trust towards the robot and acceptance of robot outcomes.

In a socially assistive task like conveying treatment and disease information, listening skills and conversational capabilities are fundamental to developing a relationship and establishing a therapeutic alliance with the patient; in this context, social robots are seen as a potential solution within these tasks (20). Previous research has shown that social robots were often viewed as more effective than computers and avatars for helping individuals track their dietary behaviours, as people tend to establish stronger relationships with them (21). Interactions with robots were also perceived more positively than with tablets, with individuals reporting greater trust in robot-delivered health instructions in Mann et al. (22). Similarly, elderly patients prefer physically present robots over virtual agents as exercise coaches (23). These previous studies demonstrated that robots' physical presence, gestures, and ability to share the patient's environment could provide advantages that 2D systems cannot replicate. Also, in the present study we are particularly interested in patients' interest for- and interaction with- a 3D entity.

In conveying treatment and disease information, it is important to consider both the robot's appearance and its ability to communicate effectively with the patient. These factors influence the patient's acceptance of the robot and their willingness to follow its instructions. Indeed, a human-like appearance alone is not a sufficient condition to increase robot acceptance. Robots must look human and act like humans (24). The robot must convey its competence and warmth to the patient in order to be trusted (15). Research shows that integrating empathetic statements from robots, such as soliciting patient feedback or expressing understanding, can significantly enhance their perceived trustworthiness. Indeed, patients who perceive robots as empathetic are more inclined to adhere to the recommendations provided by these robotic entities, ultimately improving their satisfaction with the treatment (25).

The basic idea of these dialogue-based robotic systems is that they talk and listen to end users, while providing a social (face-like) interface and provide speech-to-text and text-to-speech capabilities for natural interaction. When communicating, variants like the Furhat robot (26) place emphasis on its facial looks and on lipsync to promote a natural, speech-based interface. Furthermore, front camera's allows person tracking and basic emotion recognition, to follow the conversational partner and react based on its emotional state.

Furhat is a humanoid robotic head specifically designed for social interactions (26). The back-projected 3d face engine allows for rendering dynamic facial expressions and lip-syncing, improving the quality of the conversations and introducing all the nonverbal behaviour that might support information exchange with the patient. This is achieved through a beamer that projects face-like animations on the inside of a semi transparent plastic face mold. The robot contains a phased array microphone, speaker, and servos to operate as its neck and face muscles. Combined, these possibility of controlling gestures, neck movements, and facial expressions made Furhat a suitable choice for our study.

In terms of programming, traditional robots run script-based design time solutions, which allow them to perform pre-purposed tasks, but they can nowadays also be linked to other ICT systems (such as an electronic health record or internet-based sources), or to Large Language Models (LLMs) via an Application Programming Interface (API) (27). This allows for interactive dialogues without the need for complete preprogramming. In the present study, we investigate this latter approach, evaluating a social robot that provides patient information in patient dialogues, thus supporting patient communication and information exchange. In this way, it is possible to combine the social features of the robot with the conversational capabilities of LLMs. The use of LLMs like chatGPT to answer patient questions is an emerging field (28, 29). At present, while physicians generally consider AI responses to be accurate (29), patients still tend to prefer consulting doctors for treatment recommendations (28). However, this dynamics may shift with the integration of social presence into LLMs through the use of social robots. To our knowledge, this study is the first to explore the intersection of LLM capabilities with the social features of robots in the context of providing treatment information to OA patients.

## Materials and methods

### Social robot

We used the social robot “Furhat” (26) and linked it to a Generative Pre-trained Transformer (GPT) LLM (gpt-3.5) via an API using the Furhat Kotlin-based programming framework. To ensure conversational relevance, content accuracy, and to prevent for hallucinations, we limited the scope of the LLM to use only specific dependable medical websites whitelisted by the rheumatology department of the Medisch Spectrum Twente (MST) hospital, Enschede, the Netherlands. These websites contained physician-checked and relevant patient information on treatment options, conditions and disease management of OA. Using direct text search, these whitelisted websites are used according to the used prompt. The prompt is added in Supplementary Material A.

**Figure 1:**
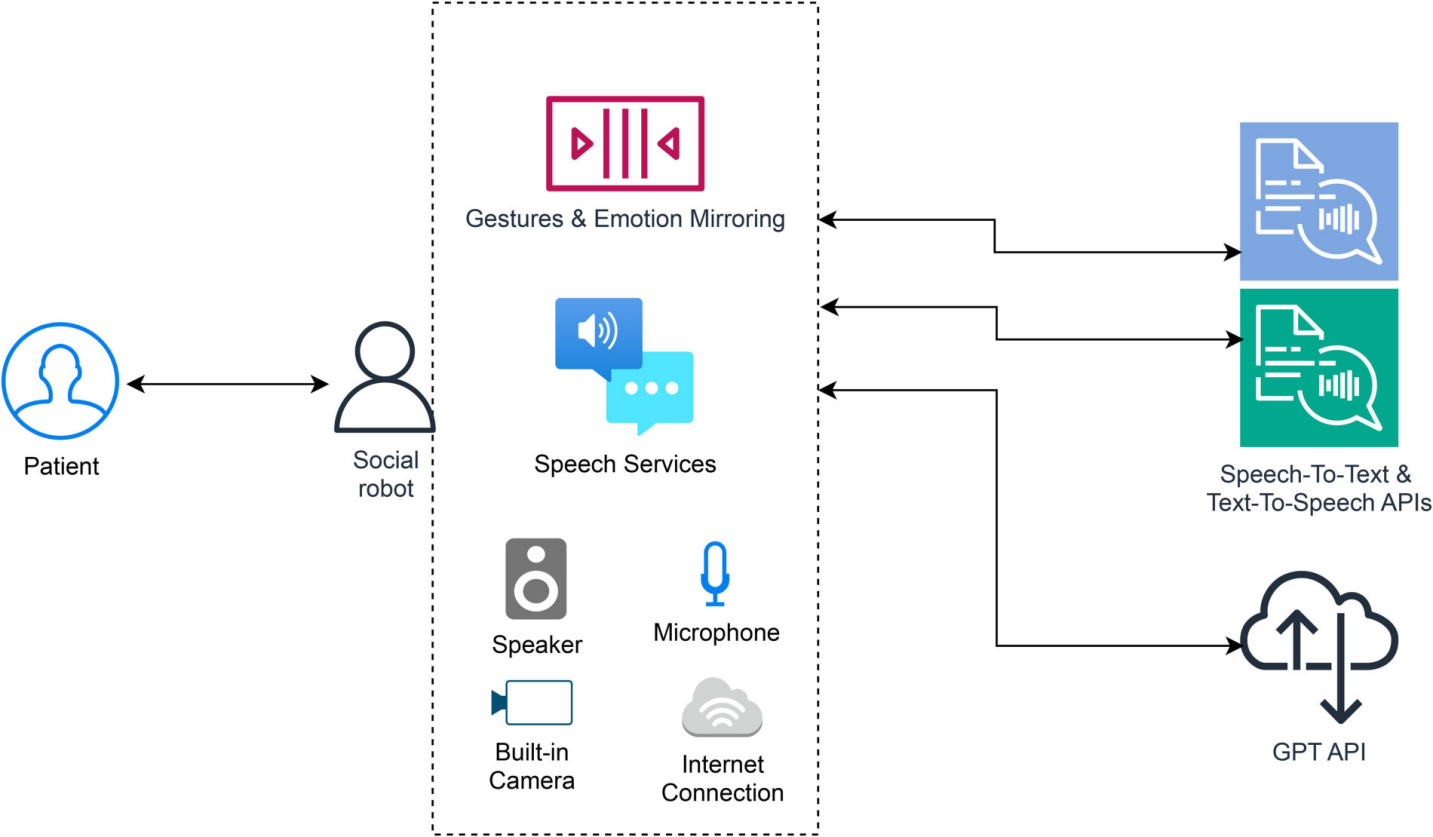
GPT-reinforced social robot architecture with robot main software and hardware components shown in dashed area. Patient talks to social robot. Social robot has built-in gestures, steering its facial expression and face movement. Using its built-camera and built-in emotion recognition, it can look the patient in the eyes, follow their movement and convey basic emotions. Its built-in speech services rely on its microphone, Amazon Polly (Text-to-Speech) and Microsoft Azure Speech Services (Speech-to-text). These are called via API endpoints and hence depend on internet connection of the robot. Once a question posed by the patient is transcribed, the GPT model is called using the prompt of Supplementary Material A to look up the answer on whitelisted medical websites. It is then sent back to the robot, and then spoken out by the robot using its builtin speaker. Created using draw.io, licensed under Apache License 2.0.

The information flow and robot components are shown in Figure 1. It entails that (in this case) a single patient talks to the social robot, after which a speech-to-text (S2T) process is triggered, relying on cloud-based recognition (Microsoft Azure Speech Services). The resulting text-string is sent via the API to the GPT, which searches for relevant answers within the whitelisted web-based data sources detailed in Supplementary Material A. The returned answer is spoken out by the robot utilizing its text-to-speech (T2S, based on Amazon Polly speech synthesis services) and Furhat lip-sync speech services.

In addition, using a basic emotional state recognition feature running on top of the person-tracking built-in camera feed within the robot, and mirroring the recognized emotion, the robot is able to track the patient's face and be triggered to convey the same basic emotion (anger, disgust, fear, happiness, sadness and surprise) as seen in the patient while talking, listening, or waiting, for better personalization. This behaviour allows for non-deterministic and more rich user interactions. The robot could potentially distinguish among multiple users and track the latest speaker, but in this study these features were not utilized.

### Experiments

First, a lab-based pilot study was performed at the Behavioural, Management and Social sciences (BMS) lab at the University of Twente, Enschede, the Netherlands, to assess the task performance and user experience of utilizing the social robot as a communication device for medical conversational purposes.

Twenty participants were enrolled to enter in a short simulation dialogue with the social robot on medication adherence, after which the User Experience Questionnaire (UEQ, 30) was filled in. The UEQ is an end-user questionnaire to measure user experience quickly in a simple and immediate way, while covering a comprehensive impression. It uses a seven-stage scale to reduce the well-known central tendency bias for such types of items, e.g., attractive—unattractive. The scale combines both ergonomic (e.g., goal or task orientation of interface) and hedonic (e.g., design originality, aesthetics of interface) aspects. UEQ is frequently used for the assessment of social robots and, together with *post-hoc* semi-structured interviews, it represents a suitable method for the nature of this study (10, 31, 44).

As a second step, feasibility was tested in actual clinical practice.

Patients visiting the outpatient rheumatology department at Medisch Spectrum Twente (MST) hospital in Enschede (The Netherlands) with either a new or an established diagnosis of osteoarthritis (OA), as well as their HCPs, were invited to interact with the social robot and to evaluate their interaction. The interaction was guided by a delineated list of potential conversation topics.

This list of questions was defined together with the treating rheumatologists.

In discussion with the rheumatology department, also the questions and answers (i.e., what the robot can talk about in respect to OA) were assessed and approved. This assessment is further detailed in van Gorssel (7).

Consent from the hospitals’ ethics and material committee was obtained (K24-22). The lists of questions, answers, surveys and robot details are provided in the Supplementary Material.

The robot started the conversation with some small talk while introducing itself and even included a small joke (“I am a social robot, the best one that you will meet today”). Then, the conversation (cf. Supplementary Material A) started as implemented. After interacting with the robot, participants completed the short UEQ (30) to assess the robot's usability and functionality in a time-effective way. The mean scores for each dimension were interpreted using standard benchmarks from UEQ studies. Scores above +1.5 were considered to reflect a positive user experience, while scores closer to 0 indicated a neutral experience. UEQ wa followed by semi-structured interviews to add in-depth opinions, perceptions and qualitative data. A control group was not used, as this is a first study to assess usability, user experiences and usefulness. Nevertheless, UEQ was chosen as a metric that allows benchmarking and comparison between patients and HCPs, and with other interaction technologies.

## Results

### Pilot test

**Figure 2:**
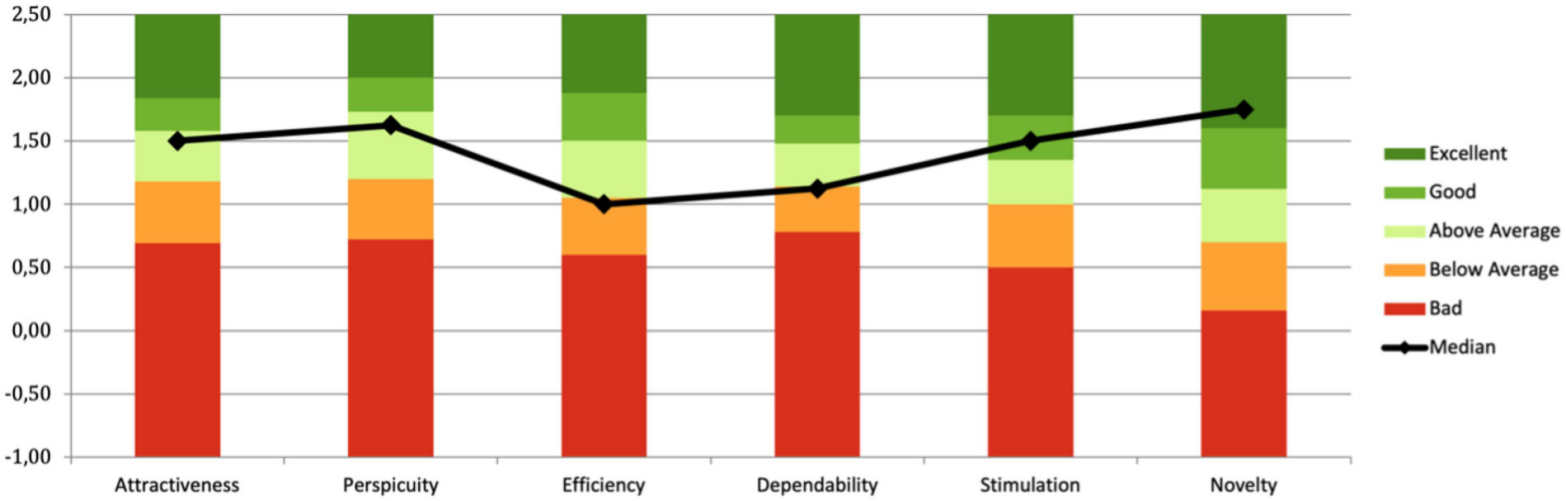
User experience questionnaire results with their component breakdown. Black line indicates the median; the traffic-light color coding indicates how the resulting scores (on *y*-axis), from low values (red) to very good values (green), compare to the UEQ benchmark (30).

In the lab-based pilot study, 10 (50%) men and 10 (50%) women (age range 20–55 years old, both workers as well as students) from various countries and cultural backgrounds including Canada, Aruba, The Netherlands, Poland, Germany, Russia, Italy, and India, participated. The setting was a 20-minute simulation conversation with the subject, robot and researcher present, after which a User Experience Questionnaire (UEQ) was filled in. The UEQ results are shown in Figure 2. Overall, above average/good attractiveness, perspicuity and stimulation were reported; novelty was excellent, yet dependability and efficiency were reported below average.

Regarding attractiveness, the mean value of 1.50 (1.17; 2.08) resulted above average. Similarly, in the perspicuity scale, the mean score of 1.63 (1.25; 2.31) indicated above-average performance. However, in the efficiency scale, the average score of 1.00 (0.75; 1.81) indicates performance within the average range. Similarly, in the dependability scale the mean score of 1.13 (0.94; 1.56) is within the average range. In the stimulation scale, the mean score of 1.50 (1.0; 2.31) indicates a good result. Finally, in the novelty scale, the average score of 1.75 (0.94; 2.3) represented a good result.

**Figure 3:**
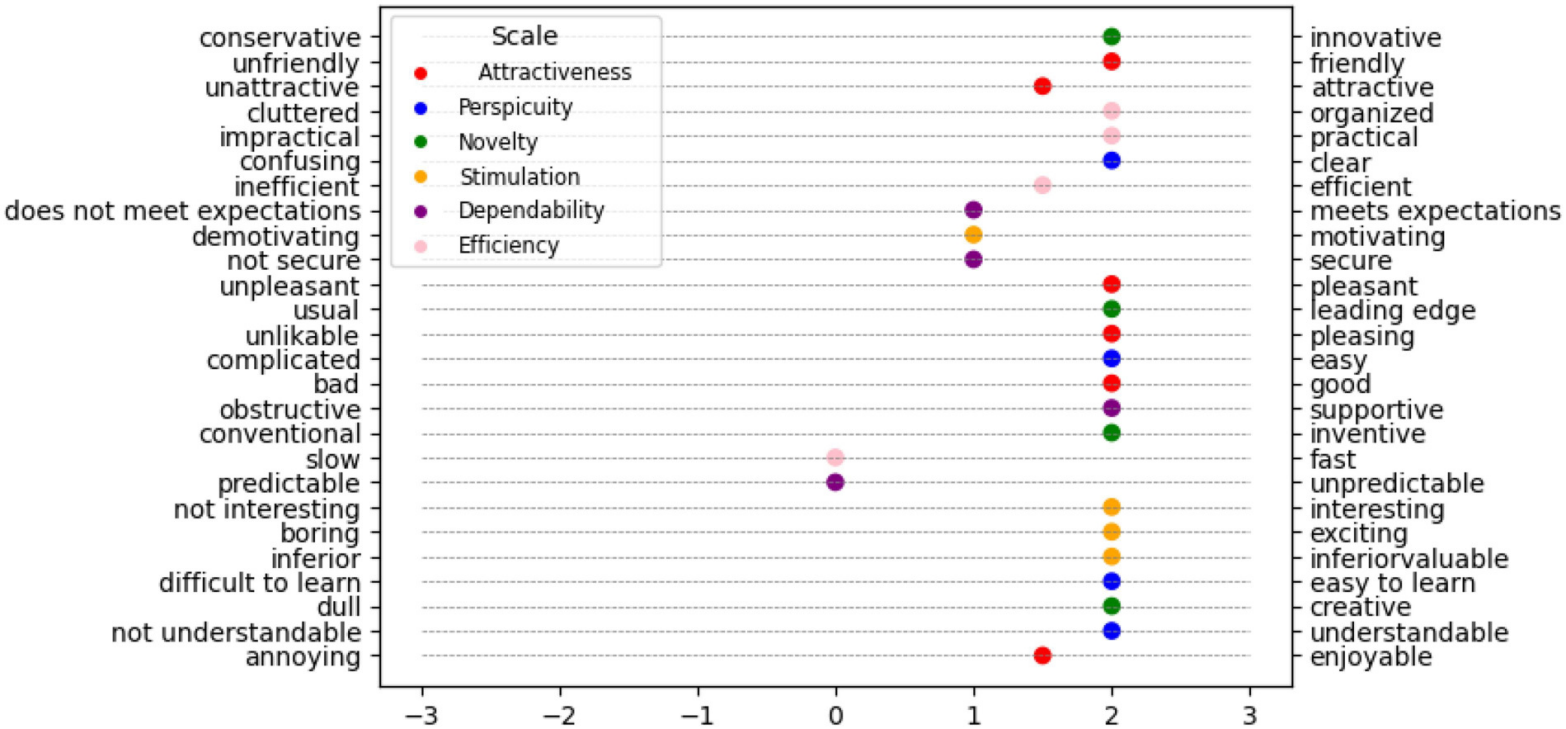
Breakdown plot of the results obtained by the user experience questionnaire item.

Figure 3 displays the UEQ item breakdown. It can be noticed how the robot's reaction time and predictability could be improved.

### Clinical practice test

Before conducting the clinical practice test, the reaction time issue (reported in the pilot test) was investigated. It was caused by the delay that occurs in checking transcribed speech to ChatGPT's API and waiting for the result; it was addressed by having the robot pronouncing an in-between response (i.e., “I will think about it.”) to bridge the time waiting for the GPT response. Also, wireless (wi-fi) connection was changed to wired connection. In addition, in the clinical test, a faster operating API endpoint as compared to the pilot test was available (gpt-4o), which resolved this issue and did not pose any barriers in patient trust.

The clinical practice test included 21 osteoarthritis (OA) patients and 7 HCPs (41).

Patients were aged in the range 43–77 years with (self-reported) mixed technology experience (from none to “a lot”) and educational level ranging from primary education to university bachelor degree level. Duration of illness ranged from “Newly diagnosed” to “Over 35 years”, and (common) comorbidities included cardiovascular diseases and diabetes.

HCPs were in the range 22–63 years old with predominantly “a little” self-reported technology experience (only two reporting “a lot”), and in professional capacity of Rheumatologists, rheumatology trainees, nurse practitioners, and medical researchers with professional experience ranging from 0 to 2 to over 6 years.

**Figure 4:**
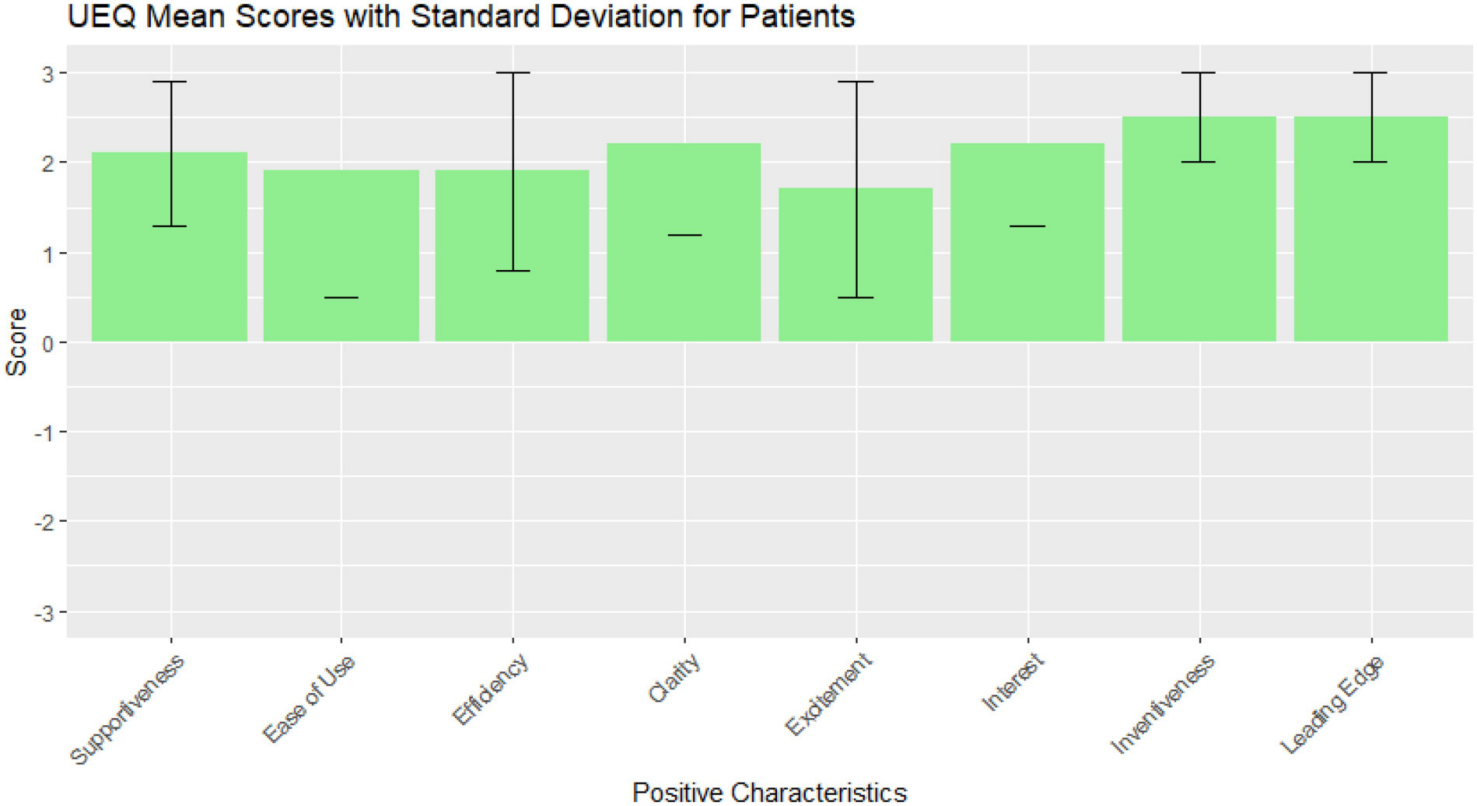
UEQ data overview for patients. Green bars represent mean value per item (between −3 and 3) with error bars indicating the standard deviation.

**Figure 5:**
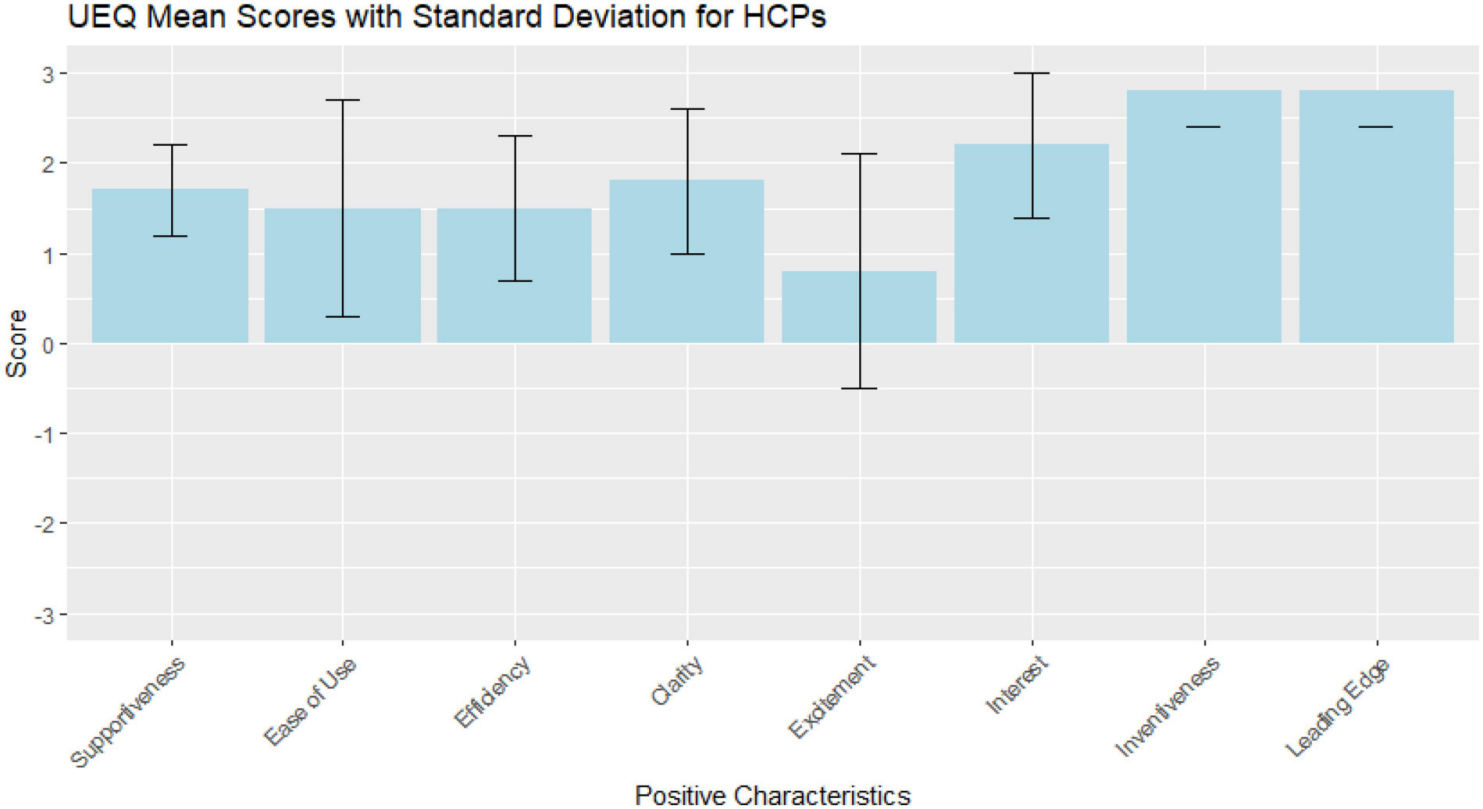
UEQ data overview for HCPs. Blue bars represent mean value per item (between −3 and 3) with error bars indicating the standard deviation.

Patient UEQ mean score (30) (Figure 4) was 2.13 with values ranging from 1.7 to 2.5, indicating a positive trend in efficiency, inventiveness and acceptability. HCPs UEQ mean score (Figure 5) resulted in 1.89, with all values above 1 except for item 5 (excitement of usage), that scored 0.8 (SD 1.3). In the interviews, patients generally found the Social Robot both acceptable and useful in a clinical setting and appreciated the robot's ability to provide information and respond to their questions, but suggested that the robot's communication style might need to be adjusted for different educational levels. For example, one participant noted “*Yes, I think it is suitable for explanation*”, and another mentioned “*If you look at conversational techniques and checking if people understood the message, improvements to the current version are possible*.”

HCPs viewed the robot as both acceptable and useful, particularly in areas such as patient education, triage, and routine follow-up, but expressed concerns about the robot's ability to replace human interaction, particularly in contexts where empathy and personalized care are crucial. One HCP remarked in the interview: “*Yes, it gave good treatment options and a clear answer*.”, but also 2 HCPs noted: “*She gives quite a lot of advice in a single sentence, sometimes mentioning 4 or 5 things*” and “*The pronunciation isn't always entirely accurate. Sometimes technical terms can be improved*”.

Patients and HCPs generally found the information provided by the robot to be both accurate and relevant, giving additional comments on communication effectiveness, engagement, personalisation of interaction (“one can ask whatever she/he wants”), perceived usefulness, emotional comfort, trust, ease of use, interaction quality, accessibility, learnabilty, error tolerance, health literacy, behaviour change ethics and care continuity. A complete coding of the *post-hoc* interviews is shown in Supplementary Material B.

**Figure 6:**
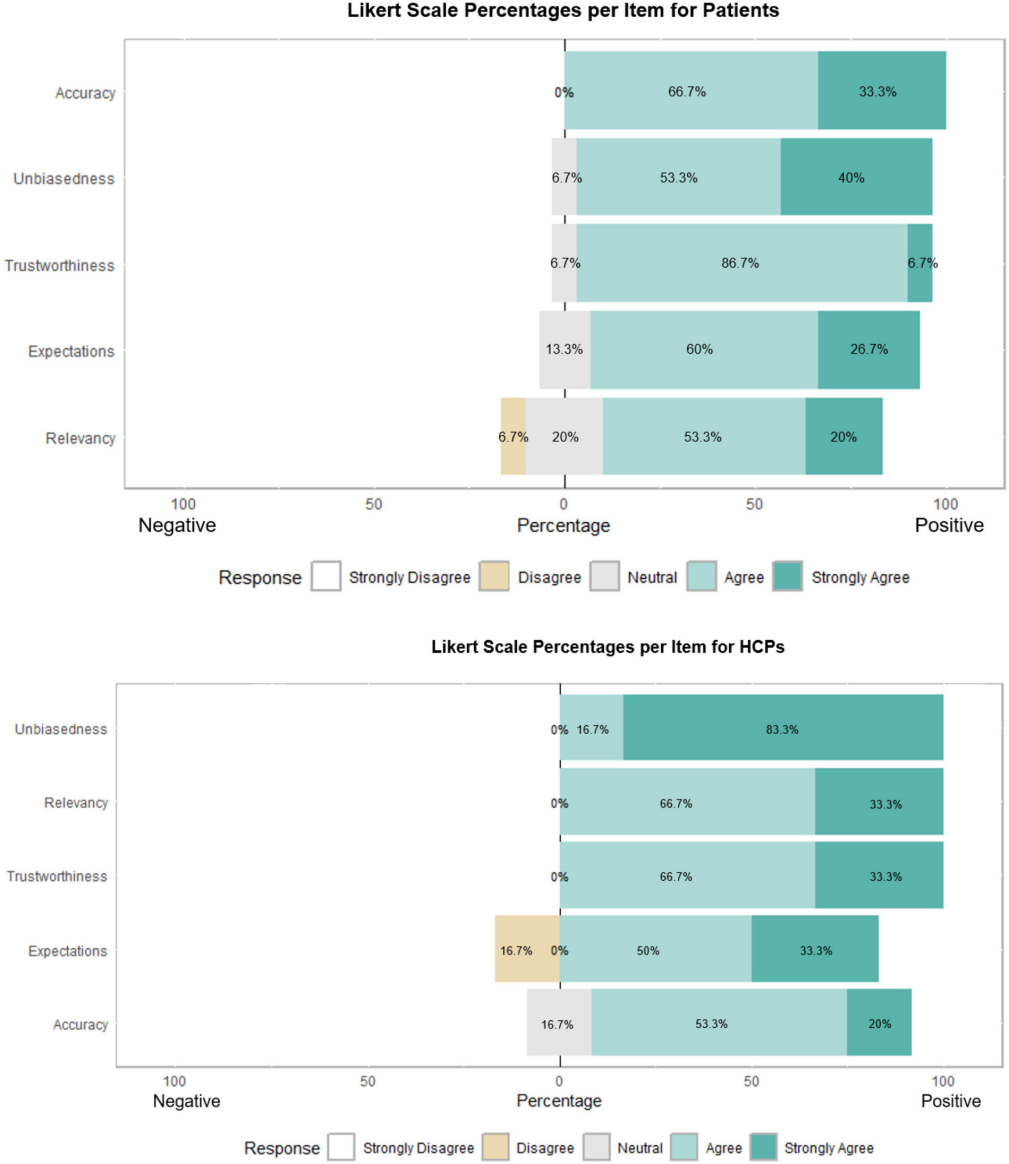
Results plot for both patients (top) and HCPs (bottom) relevant to post-usage on unbiasedness, relevancy, trustworthiness, expectations being met, and perceived accuracy of the robot.

Figure 6 shows that both patients (top) and HCPs (bottom) are mildy positive about the unbiasedness, relevancy, trustworthiness, expectations being met and perceived accuracy of the robot, with few more reservations regarding relevancy in patients, and meeting of expectations in HCPs.

## Discussion

The conducted lab evaluation and testing in clinical practice demonstrated that sufficient usability and acceptability could be reached in implementing a social robot in a clinical setting. The implementation in clinical practice resulted in a better perceived efficiency compared to the lab scenario. Both scenarios indicated general usefulness, and potential for hospital-based use in patient care. As Patient UEQ score resulted in mean 2.13, with values ranging from 1.7 to 2.5, a positive trend in efficiency, inventiveness and acceptability was noticed.

A direct comparison could be made with Gerłowska et al. (31) RAMCIP robotic assistant for older adults with mild cognitive impairments, and SERMO, a mental-health chatbot (32). Both studies employed the full UEQ scale. RAMCIP obtained median UEQ values between 0.63 and 2.0 across subscales, with particularly low results for Novelty and Efficiency (0.63–1.25), and moderate results for Attractiveness and Dependability (1.66–2.0). Similarly, the SERMO mental-health chatbot showed good pragmatic quality but neutral or low hedonic quality scores (Stimulation 0.298; Novelty 0.524) in its UEQ evaluation. In contrast, our clinical study yielded consistently higher mean scores across all UEQ subscales, for both patients and healthcare professionals. While RAMCIP users reported neutral usability due to slow interaction and developmental limitations, participants in our clinical test did not report such issues once latency was resolved. These differences suggest that combining embodiment with LLM-based conversational capabilities can enhance clarity, perceived usefulness, and engagement compared to both traditional social robots and screen-based conversational agents. The latter may be more cost-effective, but less embodied and social.

A key aspect in using LLMs is the prevention of hallucination; the implemented approach confronted this aspect by using only whitelisted sources, and this increased acceptability by the healthcare staff.

Nevertheless, patients were not tested on knowledge, so a summative rather than a formative effectiveness assessment still needs to take place. Also, a further study should compare the physical social robot approach with others, e.g., chatbot based approaches. An interesting dimension is that of trust, to consider which factors influence the establishment of trust, its breaching or recovery while using the technology (13).

Finally, privacy, security and personalisation remain important aspects for future research in the use of LLMs for clinical patient communication, including:

Data Privacy and Confidentiality: LLMs may process sensitive personal health information, raising concerns about compliance with regulations such as the Health Insurance Portability and Accountability Act (HIPAA) in the U.S. or the General Data Protection Regulation (GDPR) in the EU (33).
Data Ownership and Control: when (third-party) LLM services are integrated, it has to be clear who owns the patient data or how it may be reused or stored, raising legal and ethical concerns (33).
Model Memorization and Information Leakage: LLMs have demonstrated to memorize parts of their training data, which could lead to the unintentional disclosure of sensitive patient information during future interactions (34), although in the present system these cannot be linked to specific individuals.
Informed Consent Challenges: patients may not be fully aware that an AI system is involved in the communication process, or may not understand how their data is used or stored, thus compromising ethical standards for informed consent (35).
Security Vulnerabilities in System Integration: integrating LLMs into electronic health record systems could introduce new cybersecurity threats, particularly through additional (insecure) APIs or improper configuration (36).
Over-Reliance and Misuse: although verified beforehand in the present case study, clinicians may overestimate the reliability of LLMs in general, leading to over-sharing of information or uncritical adoption of AI-generated advice (37).
Data Retention and Logging: many LLM services log user interactions which, if not anonymized or protected, could lead to unauthorized access or secondary misuse of health data (33). Hence, system security should be carefully examined when implemented in the hospital.
Transparency and Auditability: due to their black-box nature, LLMs often lack explainability, making it difficult to trace decisions, detect errors, or assign accountability (38). This could be partially resolved by using whitelisted clinical information sources only and standardization, but explainability remains a key aspect even then.
Availability: although response speed and availability did not pose any barrier in patient trust in the current study, latency in using LLMs could represent a limitation and should be improved in future work.
Personalisation: by personalisation towards the end users, a higher level of acceptance, privacy, and confidentiality could be achieved, beyond allowing a patient to ask anything he/she wants. This paper also explored mirroring as a personalisation technique, but the access to the information included in the personal electronic health record (HER), and tailored answers based on unique users' questions, should also be explored as personalisation directions.

## Conclusion

This paper demonstrated the initial feasibility of implementation of a GPT-reinforced social robot in clinical practice, such as supporting treatment and disease educational communication for patients with osteoarthritis. Patients with rheumatic diseases see the AI-reinforced Social Robot as a potentially valuable tool for providing information and supporting patient education. Healthcare professionals appreciated the innovation introduced and could recognize its future potential, with emphasis on a complementary rather than replacing role. Hence, the need for human oversight remains.

Despite these promising results, several areas require improvement before large-scale deployment. Personalisation was intentionally limited in this study; however, future implementations will need to explore how robotic conversational strategies can be tailored to individual patient characteristics. Evidences from previous work show that personalised AI interactions play a crucial role in enhancing patient satisfaction and improving treatment adherence (39, 40). What remains unknown is whether these benefits translate to embodied AI systems such as social robots, and how personalisation should be implemented safely and effectively in this context. In a future study, we will investigate language level adaptation in order to personalize the communication by the robot through an adapted language, based on patient preferences, health literacy, and characteristics.

Furthermore, our study was conducted in a single centre in the Netherlands, with a sample of 21 patients and 7 healthcare professionals. Cultural factors, organisation of care, and levels of digital literacy may influence how an AI-enhanced social robot is perceived. Future work should therefore replicate and extend this evaluation preferably in multi-centre and multi-country settings to examine how contextual and geographical factors could shape acceptance, trust, and effectiveness. In terms of effectiveness, both economical and communicative effectiveness in terms of retention of information) should be assessed.

Finally, although trust was not negatively affected in this feasibility phase, long-term trust trajectories and possible points of breakdown between users and robots remain essential to be investigated.

This study demonstrated the possibility of providing accurate, relevant, and timely medically validated information. Future multicentre and multi-country studies, combined with the exploration of personalisation and long-term trust, will be essential to determine whether AI-enhanced social robots could reliably and safely complement human clinicians in delivering clear, consistent, and patient-tailored health information in real-world clinical practice.

## Data Availability

The raw data supporting the conclusions of this article will be made available by the authors, without undue reservation.
