# Correction: A GPT-reinforced social robot for patient communication: a pilot study

**DOI:** 10.3389/fdgth.2026.1812402

**Published:** 2026-03-03

**Authors:** Jan-Willem J. R. van ‘t Klooster, Michela Capasso, Daan van Gorssel, Elvis Vrolijk, Giorgio Rettagliata, Demy Gerritsen, Mirjam Hegeman, Emanuele Tauro, Enrico Gianluca Caiani, Harald E. Vonkeman

**Affiliations:** 1 Behavioural Management and Social Sciences, University of Twente, Enschede, Netherlands; 2 Dipartimento di Elettronica, Informazione e Bioingegneria, Politecnico di Milano, Milano, Italy; 3 Department of Rheumatology and Clinical Immunology, Medisch Spectrum Twente, Enschede, Netherlands; 4 IRCCS Istituto Auxologico Italiano, San Luca Hospital, Milan, Italy

**Keywords:** GPT, osteoarthritis, patient communication, social robot, UEQ

Erroneously assigned

Author Michela Capasso was erroneously assigned to affiliation Department of Rheumatology and Clinical Immunology, Medisch Spectrum Twente, Enschede, Netherlands. This affiliation has now been removed for author Michela Capasso. The correct affiliation for author Michela Capasso is Dipartimento di Elettronica, Informazione e Bioingegneria, Politecnico di Milano, Milano, Italy.

The original version of this article has been updated.

