# Supplementary material for: A GPT-reinforced social robot for patient communication: a pilot study
Source: Front Digit Health. 2026 Jan 27;7:1653168. doi: 10.3389/fdgth.2025.1653168 (PMC12887852; doi:10.3389/fdgth.2025.1653168)
Supplement: Supplementary file 1 [file Datasheet1.pdf]

Supplementary Material A – Conversation example

```kotlin
dialogues = listOf(
    Dialogue(
        id = "0",
        question = utterance{+"Voor u ziet u een lijst aan standaard vragen."
            +Gestures.Nod
            +"Neem u tijd om deze lijst rustig door te lezen. Als u klaar bent zeg dan: ik ben klaar."},
        answers = listOf(
            Answer( userInput: "Ik ben klaar", utterance{+"Perfect"
                +Gestures.Wink}, followUps: "1"),
            Answer( userInput: "Ja", utterance{+"Perfect"
                +Gestures.Wink}, followUps: "1"),
            Answer( userInput: "Ik ben niet klaar", utterance{+"Is goed, neem uw tijd."}, followUps: "0"),
            Answer( userInput: "Nee", utterance{+"Is goed, neem uw tijd."}, followUps: "0"),
        )
    ),
    Dialogue(
        id = "1",
        question = utterance{+"U kunt een nummer van een standaard vraag opnoemen en dan zal ik deze beantwoorden. Als u
        answers = listOf(
            Answer(
                userInput = "Andere vraag",
                response = utterance{+"U heeft gekozen voor een andere vraag."},
                followUps = "chatGPT",
                redirect = "4"
            ),
            Answer( userInput: "1", utterance{+"Artrose is een vorm van reuma. Dit kan voorkomen in 1 of meerdere gewrichten
            Answer( userInput: "2", utterance{+"Ondanks veel onderzoek is onbekend hoe artrose ontstaat. Wel zijn er verschi
```

Figure A.1. Kotlin excerpt with example of how the dialogues are structured. The robot first points at a list of standard questions. If the patient mentions he/she is ready, it allows answering standardized questions, or other questions.

Prompt used:

Zoek alleen voor informatie op de volgende websites: reumanederland, zorginzicht, reade.nl, nvr.nl/richtlijnen, poly-artrose, medipoint en able2. Het antwoord moet gegeven worden aan een patient, dus hou het kort, bondig en simpel.

Which translates as:

Only look for information on the following websites: reumanederland, zorginzicht, reade.nl, nvr.nl/richtlijnen, poly-artrose, medipoint en able2. The answer must be given to a patient, so keep it short, concise and inelaborate.

Supplementary Material B – Interview results

Table B.1 Interview transcripts from all patients (Table B.1) and HCPs (Table B.2) coded and explained

**Main code**	**Sub Codes**	**Examples from transcripts**	**Explanation**
User-Robot interaction	Communication effectiveness	- "Ze geeft best veel advies in een zin, soms noemt ze wel 4/5 dingen."	Communication effectiveness was impacted by the robot's tendency to provide too much information quickly or elaborate

		- "De robot praatte wel snel maar wel duidelijk."<br>- “Soms wordt het wat uitgebreid en worden er dingen bijgehaald."<br>- "Technisch was het zeer goed te volgen, maar er zijn nog verbeterpunten."	excessively. While technical aspects were good, improvements in response modulation and handling of complex terms could enhance effectiveness.
	Engagement	- "Heel positief, vooral heel wonderlijk."<br>- "Ja, zeker wel plezierig."<br>- "Ik vond het leuk om te doen, ondanks de kunstmatige elementen."<br>- "Het was een leuk idee, vooral met de grappige elementen in het begin, maar het kan nog veel verbeteren."	Engagement was high, with the interaction being generally enjoyable. Users appreciated the robot’s friendly demeanor and human-like features, though improvements in response variety and naturalness were suggested.
	Personalization of interaction	- "Je kunt vragen wat je wilt."<br>- "Het zou wel fijn zijn om door te kunnen vragen op het onderwerp dat we aan het bespreken zijn zonder interruptie."<br>- "Ja, als je diagnose met lichamelijk onderzoek hebt gehad, dat er sprake is van artrose,	The ability to ask various questions and the potential for personalized responses were seen as positive aspects. There is interest in integrating the robot into different stages of patient care and providing personalized summaries of discussions.

		dat het dan verder gaat via de sociale robot."<br>- "Het zou fijn zijn als de robot kan herinneren wat eerder besproken is en dat er een samenvatting aan het eind komt."	
Acceptability	Perceived usefulness	- "Ja, ik denk dat het zeker waard is om te proberen. Ik denk dat dit een goede manier is van informatie verstrekken."<br>- "Heel positief, vooral heel wonderlijk."<br>- "Ja, ik denk dat het voor uitleg zeker wel geschikt is."<br>- "Als je kijkt naar gesprekstechnieken en checken of mensen goed die begrepen hebben, zou dat een verbetering op de huidige versie zijn."	The robot is seen as a useful tool for providing information. Its potential to enhance information delivery and the desire for improvements in communication techniques highlight its evolving utility. Participants mentioned a reflection tool, also repeating given information, would help in overall usefulness.
	Emotional Comfort	- "Ja, zeker wel plezierig. [...] Af en toe ook een beetje fake glimlach."<br>- "Het was leuk al die menselijke uitingen."<br>- "Ja, jawel, ik vond het leuk om te doen, alleen de kunstmatige pauzes	The interaction was generally pleasant, though artificial elements like pauses and expressions sometimes impacted emotional comfort. Users appreciated human-like features but suggested improvements for a more natural end to the conversation.

		waren een beetje storend."<br>- "Het eind mag vriendelijker en minder abrupt zijn."	
	Trust in information	- "Ja, de informatie was wel nuttig."<br>- "Ja, het is echt een vraagbaak, heel vriendelijk is ze."<br>- "Ja, het gaf goede opties qua behandeling en op zich helder antwoord."<br>- "Technisch was het zeer goed te volgen."	The robot's information was considered reliable and helpful. Trust in the robot's guidance was high, with clear and accurate responses enhancing its credibility.
Usability	Ease of use	- "Ja, ik vond het goed te volgen, wel vond ik dat ze een beetje vlot praat."<br>- "Het gesprek met de robot was ook goed te volgen."<br>- "Ja, het zijn op zich wel redelijk goede antwoorden antwoord op je vraag."<br>- "Technisch was het zeer goed te volgen."	The robot was generally easy to follow, though the fast pace of its speech and complex terminology could be challenging for some users. Technical aspects of its usability were praised, with some room for improvement in clarity.
	Interaction quality	- "De standaardvragen voelde heel kunstmatig."<br>- "De robot praten wel snel maar wel duidelijk	Interaction quality was affected by the robot's tendency to provide too much information quickly or to elaborate excessively. Users appreciated

		en de informatie was ook duidelijk."<br>- "Soms wordt het wat uitgebreid en dingen bijgehaald, maar niet te lang."<br>- "Er mag nog wat verbetering komen in de modulatie van antwoorden."	clarity but suggested improvements in the robot's response modulation and variety to enhance interaction.
	Accessibility	- "Ja dat zou ik wel vinden. [...] zolang er ook een verpleegkundige is zodat je verder uitleg kan krijgen."<br>- "Het is een goede manier om extra informatie te geven."<br>- "Ja, ik denk dat het op zich wel kan."<br>- "Het is enorm acceptabel in een polikliniek, vooral omdat iedereen haast heeft en de robot alle tijd heeft."	The robot is seen as a useful tool for providing accessible information, with the potential to improve the patient experience in a busy clinical setting. It is appreciated for its capacity to offer information without time constraints, though human support remains important for complex needs.
	Learning curve	- "Misschien in het vervolg mensen adviseren om niet al te lang in die standaardvragen te blijven hangen."	Recommendations included simplifying interactions and focusing on open-ended questions to ease the learning curve. Improving the robot's response variety and modulation could enhance

		- "Ja, dan denk ik dat dat fijn is."<br>- "Ja of een ander zinnetje en na een paar keer snap je het concept."<br>- "Er mag nog wat verbetering komen in de modulatie en variatie van antwoorden."	understanding and user comfort.
	Error tolerance	- "Ze geeft best veel advies in een zin, soms noemt ze wel 4/5 dingen."<br>- "Ja, de informatie was ook gemakkelijk te begrijpen."<br>- "De uitspraak klopt inderdaad soms ook nog niet helemaal."<br>- "Soms zijn er technische woorden die verbeterd kunnen worden."	The robot's tendency to provide too much information at once or use complex terminology sometimes made it harder to process. Pronunciation and technical jargon issues were noted, indicating areas for improvement in error tolerance and clarity.
Impact on patient outcomes	Health literacy	- "De uitspraak klopt inderdaad soms ook nog niet helemaal."<br>- "Soms zijn er technische woorden die verbeterd kunnen worden."	The robot's handling of medical terminology could be improved. Some users found certain terms and pronunciations confusing, indicating a need for clearer explanations and more accurate language use.
	Behavior Change	- "Het zou helpen om aan gedragsverandering te werken, zoals het volgen	The robot has potential to influence health behaviors by providing motivating

		van een dieet of medicatie-instructies."<br>- "Het biedt goede informatie die mensen kan motiveren om gezondere keuzes te maken."<br>- "Het kan patiënten helpen bij het zelfbeheer van hun gezondheid door duidelijke instructies te geven."<br>- "Ja, de robot biedt praktische ondersteuning voor zelfmanagement."	information and instructions. Users recognized its role in supporting adherence to health-related behaviors and decisions.<br><br>The robot supports self-management by offering practical advice and clear instructions. This helps patients manage their health more effectively and adhere to recommended practices.
Ethical and privacy concerns	Ethical concerns	-	None were mentioned in the interviews
Integration into Healthcare	Continuity of Care	- "Het zou wel kunnen, denk ik, ja."<br>- "Het is inderdaad een goede aanvulling als eerste informatie."<br>- "Ja, ik denk dat het voor uitleg zeker wel geschikt is."<br>- "Het is enorm acceptabel, vooral omdat de robot alle tijd heeft en patiënten kunnen veel vragen stellen."	The robot is viewed as a valuable tool for enhancing continuity of care, particularly as a resource for providing information post-consultation. It is seen as a useful addition to the healthcare process, improving patient access to information.

Table B.2 Interview transcripts from all patients coded and explained

**Main code**	**Sub Codes**	**Examples from transcripts**	**Explanation**
Patient-Robot interaction	Communication effectiveness	- "Het gesprek met de robot was duidelijk en de antwoorden waren goed te begrijpen."<br>- "De instructies waren helder en makkelijk te volgen."<br>- "Ik vond het prettig dat er geen moeilijke woorden werden gebruikt."	The robot was generally effective in communication, with clear and understandable responses. Patients found the language simple and the instructions easy to follow, which contributed to a positive interaction experience.
	Engagement	- "Je hebt er geen band mee."<br>- "De relatie met de robot was positief, vooral vanwege de duidelijke antwoorden."<br>- "Voor ouderen kan de robot een goede aanvulling zijn, hoewel het geen vervangende menselijke interactie biedt."	Engagement varied, with some patients missing the emotional connection, while others appreciated the robot's knowledge and clarity. The robot's positive impact despite the lack of human contact was noted, and it was seen as particularly beneficial for older patients.
	Personalization of interaction	- "De gezichtsuitdrukkingen van de robot waren een beetje streng,	The robot's interaction was perceived as somewhat personalized through its facial

		maar ook vriendelijk op andere momenten."<br>- "Soms vond ik dat de robot me meer persoonlijk kon aanspreken, maar het blijft een machine."<br>- "Ik vond de robots stem vriendelijk, wat de ervaring persoonlijker maakte."	expressions and tone of voice, although it is still limited by its machine nature. Patients appreciated the friendly tone and the robot's ability to adjust its expressions, but they recognized that true personal connection was lacking.
Acceptability	Perceived usefulness	- "Er wordt informatie gegeven, maar dat zijn vaak dingen die je al weet."<br>- "Het gesprek met de robot was zeer informatief."<br>- "Ik denk dat ik daar eerst zelf verder voor zal zoeken."<br>- "Als je daardoor sneller antwoord krijgt op je vragen."<br>- "Ik vind het verrassend positiever dan ik verwacht had."<br>- "Voor ouderen is het ideaal, geeft goede informatie en voegt iets toe."	Patients generally found the robot useful for providing information, though some felt it didn't add much beyond what they already knew. Many found the robot surprisingly positive, offering a valuable addition for answering questions. The robot was also seen as particularly useful for older patients.

	Emotional Comfort	- "Je hebt geen menselijke warmte."<br>- "Heel goed te volgen. Ik vond het heel duidelijk."<br>- "Ik vond het wel spannend, dat wel, maar toch wel plezierig."<br>- "De stem is vriendelijk. Het hele gesprek was gewoon wel vriendelijk en rustig."<br>- "Het gevoel dat je kan delen met de robot, hoewel het een machine is, was aangenaam.	The robot was seen as clear and easy to follow, but some patients missed human warmth and found the interaction somewhat impersonal or unnerving. The robot's friendly and calm demeanor was appreciated by some, and it provided a sense of comfort despite being a machine
	Trust in information	- "Het was toch wel allemaal informatie die al bekend was."<br>- "Nee, heel neutraal, objectief."<br>- "Nee dat heb ik niet gemerkt," (in response to whether the robot was trying to sell anything).<br>- "De informatie was ook neutraal, zonder oordeel."	Trust in the robot's information was generally high, with patients finding it neutral and unbiased. Some found the information not novel but applicable and beneficial.

		- "Ja, de informatie was nuttig en goed toepasbaar."	
Usability	Ease of use	- "Het gesprek was goed te volgen."<br>- "Nee, ik vind hem heel duidelijk."<br>- "Helemaal goed," (when asked about the language used by the robot).<br>- "Het was goed te begrijpen, en antwoorden waren rustig en duidelijk."<br>- "Stem was duidelijk, techniek werkte goed, antwoorden waren snel en begrijpelijk."	The robot was generally considered easy to use, with clear communication that was accessible to most patients. The interaction was described as straightforward and understandable, with good technical performance.
	Interaction quality	- "Het is moeilijk dat je altijd dezelfde vraag moet stellen."<br>- "Wellicht toch iets meer duidelijkheid wat je allemaal kan vragen."<br>- "Het antwoord was altijd precies op mijn vraag."<br>- "Soms was het gesprek een beetje repetitief."	There was a desire for more varied interaction and clearer guidance on what to ask. Some patients found the repetitive nature of questions a limitation but appreciated the accuracy of the answers given.

		- "Duidelijk en snel te begrijpen antwoorden, hoewel je soms niet precies wist wat te vragen."	
	Accessibility	- "Het gesprek was goed te volgen."<br>- "Technisch gezien, werkte het goed en was het gemakkelijk toegankelijk."	The robot was accessible and easy to use for most patients, with technical aspects functioning smoothly and interactions being easy to follow.
	Learning curve	- "Je weet nog niet hoe de robot reageert."<br>- "Ik denk als je een beetje een keertje vaker hebt gedaan, ik denk dat je dan wel zegt van ja, daar heb ik ook wel wat aan."<br>- "Ja, dit was de eerste keer, het was een positieve verrassing."	Participants acknowledged an initial learning curve but believed that familiarity would improve the experience. Initial surprises were positively noted, and adaptation to the system over time was anticipated.
	Error tolerance	- "Ik maakte zelf een paar foutjes, maar de robot ging daar goed mee om."<br>- "De robot gaf snel correcties en begreep de meeste van mijn foutjes."<br>- "Soms raakte ik de draad kwijt, maar de	The robot demonstrated a reasonable level of error tolerance by handling mistakes or misunderstandings effectively. It was able to correct errors and maintain the flow of conversation, enhancing the usability experience.

		robot herstelde het gesprek goed."	
Impact on patient outcomes	Health literacy	- "Als je iets niet weet, moet je dat opzoeken. Dat is soms lastig. De robot doet dit nu voor mij."<br>- "Misschien kan een menu bijdragen, dat je dan wat minder vragenlijsten hebt."<br>- "Ik moet er nu zelf over nadenken. Oh, het is wel voor de ene maar niet, zeg maar, voor andere dingen dan de artrose."<br>- "Ja, dat waren toch ook wel de vragen die je zelf wel beantwoord zou willen hebben."<br>- "De robot gaf goede informatie en voegde iets toe aan wat ik al wist."	The robot helped clarify medical information for some, potentially enhancing health literacy. It was seen as a helpful tool for addressing questions that users might have hesitated to ask and providing additional insights. Patients felt like their health literacy could increase from interacting with the robot. Some mentioned a clearer overview of what type of questions to ask.
	Behavior Change	- "Ik heb er eigenlijk weinig aan gehad."<br>- "Je moet toch eerst eens verder kijken."<br>- "Dan merk ik al wel van: ja, je hebt heel veel pijn, ja en dat	Some patients indicated that the robot's information might influence their behavior, while others felt it had little impact. The robot was considered potentially beneficial for

		moet ik zelf ook leren."<br>- "Het had invloed op wat ik wilde weten over mijn gezondheid."<br>- "Voor ouderen kan het helpen om vragen te stellen die ze anders misschien vergeten."	those who struggle with asking questions or remembering information, yet not as beneficial to patients already way ahead in their osteoarthritis patient journey.
Ethical and privacy concerns	Ethical concerns	- "Een robot kan een arts niet vervangen."<br>- "Als het alleen een robot zou zijn, dan zou ik dat minder goed vind."<br>- "Nee, het is meer, echt een extra."<br>- "De robot biedt aanvulling, niet vervanging."<br>- "Ik heb geen ethische problemen opgemerkt."	Patients generally viewed the robot as a supplementary tool rather than a replacement for human doctors, which eased ethical concerns. The robot's role was seen as supportive rather than substitutive.
Integration into Healthcare	Continuity of Care	- "Een robot kan een arts niet vervangen, maar wel een aanvulling zijn."<br>- "Het is een extra hulpmiddel dat niet de menselijke interactie vervangt."	The robot is viewed as a complementary tool rather than a replacement for human doctors. It is seen as enhancing continuity of care by providing additional information and support, but not

		- "Zou goed kunnen passen als extra informatiebron, maar moet geen vervanging zijn voor artsen."	substituting the need for personal medical consultations. The robot is seen as adding value by supporting ongoing patient care and information access without disrupting the existing care structure.
Patient Demographics and Context	Age and technology familiarity	- "Voor ouderen is het ideaal."<br>- "Misschien meer geschikt voor mensen die moeite hebben met het stellen van vragen."	The robot was considered especially beneficial for older patients and those who might struggle with asking questions. Its design and functionality were seen as well-suited to these groups.
